# Early life climate and adulthood mental health: how birth seasonality influences depressive symptoms in adults

**DOI:** 10.1186/s12889-023-15145-5

**Published:** 2023-01-31

**Authors:** Hao Zhou, Danni Peng-Li, Juan Chen, Dong Sun, Bin Wan

**Affiliations:** 1 Stem Cell Transplantation Laboratory, Ningbo First Hospital, Ningbo, China; 2 Max Planck Institute for Human Cognitive and Brain Sciences, Leipzig, Germany; 3 International Max Planck Research School on Neuroscience of Communication: Function, Structure, and Plasticity (IMPRS NeuroCom), Leipzig, Germany; 4 School of Public Health, Sun Yat-sen University, Guangzhou, China; 5 Institute of Psychology, Chinese Academy of Sciences, Beijing, China; 6 Sino-Danish College (SDC), University of Chinese Academy of Sciences, Beijing, China; 7 Department of Food Science, Aarhus University, Aarhus, Denmark; 8 Department of Occupational and Environmental Health, School of Public Health, Xi’an Jiaotong University Health Science Center, Xi’an, China; 9 School of Public Health, Peking University, Beijing, China

**Keywords:** Depressive symptoms, Birth seasonality, Interaction model, Generation

## Abstract

**Background:**

Early life in-utero can have long-term influence on the mental health status of individuals in adulthood, such as depression. Age, gender, socio-economic status, education, and geography are demographic factors shown to be particularly vulnerable towards the development of depressive symptoms. In addition, climate risks on depression include sunlight, rain, and temperature. However, whether climate factors in early life have a long-term influence on depression related to demographic vulnerability remains unknown. Here, the present study explored the association between birth seasonality and adulthood depressive symptoms.

**Methods:**

We employed data from the project of Chinese Labour-forces Dynamic Survey (CLDS) 2016, containing the epidemiological data of depressive symptoms with a probability proportional to size cluster and random cluster sampling method in 29 provinces of China. A final sample size of 16,185 participants was included. Birth seasonality included spring (March, April, and May), summer (June, July, and August), autumn (September, October, and November), and winter (December, January, and February).

**Results:**

We found that born in Autumn peaked lowest rate of having depressive symptoms (16.8%) and born in Summer (vs. Autumn) had a significant higher ratio (OR = 1.14, 95%CI = 1.02, 1.29) when controlling for demographic variables. In addition, demographic odds ratio of having depressive symptoms differed between people born in different seasons, particular for age and geography.

**Conclusion:**

Our findings suggest that birth seasonality influences the sensitive link of depressive symptoms with age and geography. It implicates early life climate environment may play a role in the development of adulthood depressive symptoms.

## Background

Depression refers to a group of mental disorders characterized by significant mood depression. Approximately 264 million people worldwide are affected by depression [1, 2] and have experienced a significant decrease in quality of daily life [3, 4]. Besides, robust evidence has shown that depression is statistically associated with various kinds of diseases [5–7]. Since the Reform and Opening in China, the living standards of residents have been greatly improved. However, a series of problems have followed during the urbanization process (e.g., narrow living space, fast-paced life, high-intensity work and heavy life pressure). Consequently, a growing number of people face the disturbance of anxiety and depression disorders [1, 8], which in turn has caused economic loss and social burden in China [9, 10].

A large body of research has linked depressive symptoms to socio-demographic factors. For example, being female (vs. male) and older (vs. younger) as well as having a lower (vs. higher) income level and education degree is followed by a higher risk of experiencing depression [11–14]. Likewise, geographic factors are also connected to depression, with individuals in Northern China being more susceptible [15]. Even, early environment in-utero can potentially form a long-term impact on human development [16–18]. In particular, numerous studies have reported the effects of birth seasonality on depressive symptoms. An early report demonstrated that major depression had an excess in spring births in the US [19]. In an Italian population, individuals born during spring or summer months were reported with higher sensitivity to seasonal changes [20]. In addition, a Polish study found that birth month might be significantly associated with the course of recurrent depressive disorders [21]. However, some other scholars found birth seasonality has no direct relation with depressive symptoms. For example, Lorenzo Tonetti et al. reported that the results were not statistically significant between birth seasonality and the personality traits of healthy adults [22].

Interestingly, although the association between demographic factors and depressive symptoms is stable, the sensitivity seems vulnerable in different populations. One study found that there were excess births from winter to early spring with mood disorders, particularly in females [23]. In the same vein, the interaction of birth month with lifestyle was found to be significant in committing suicide in a Greenlandic population [24]. Another study found that Australian individuals born in the Southern hemisphere from September to November had a higher prevalence of depressive symptoms compared to individuals born in the Northern [25]. Collectively, these studies indicate that birth seasonality can change the demographic sensitivity to mental health symptoms.

Most studies were confined to data from developed countries and with varying results from different populations. Recent evidence, however, highlighted the association between birth seasonality and schizophrenia in a large-scale Chinese study [26], yet evidence on the association between adulthood depressive symptoms and birth seasonality is still lacking. To testify such the effect, we explored the influence of birth seasonality on depressive symptoms in Chinese adults.

Further, to test whether the sensitivity of demographic characteristics to depressive symptoms is changed depending on birth seasonality, the individuals were categorized into four groups (i.e., spring, summer, autumn, and winter). Here, we hypothesized that the relationship between depressive symptoms and demographic characteristics, including gender, age, socio-economic status, education, and geography, is not stable in populations with different birth seasonality. We will study the interaction of demographic factors with birth seasonality on depressive symptoms in whole population.

## Methods

### CLDS sample

The cross-sectional data used in this paper were obtained from the China Labour-force Dynamics Survey (CLDS) held by the Centre for Social Science at Sun Yat-sen University in Guangzhou, China (please refer to http://css.sysu.edu.cn for more information about the CLDS data) in 2016. The sampling method and details were introduced in our former report [12]. For the primary sampling, the municipalities and counties were selected according to a series of criteria, such as per capita gross domestic product (GDP), demographic structure, and geographic region. The second stage randomly sampled the villages or community as secondary sampling units using the probability proportionate to size (PPS) sampling method. With participants’ spoken consent, surveyors visited their home and requested them to complete the questionnaires individually and confidentially.

21,091 individuals participated in the survey. There were no records of birth month for 3,949 individuals or no age information for 19 individuals. We also excluded 472 individuals with age below 18 years old. Besides, there were no records of the CES-D scale for 449 individuals or missing education information for 17 persons. After the screening procedure, the final valid sample size consisted of 16,185 individuals.

### Measurements

*Demographic characteristics*.

The demographic categorical variables in our analyses included gender, age, education degree, socio-economics, and geography.

- **Gender** was categorized into male and female. As listed in Tables 1, 7 and 552 (46.7%) males completed the survey.
- **Age** of individuals ranged from 18 to 83 (mean = 46.3, SD = 13.3) years. It was categorized into four groups, i.e., young group (from 18 to 30 years, N = 2,620, 16.2%), young-middle group (from 31 to 45 years, N = 4,347, 26.9%), middle group (from 46 to 60 years, N = 6,629, 41.0%), and elderly group (more than 60 years, N = 2,589, 16.0%).
- **Education** as “the highest level of education obtained” was differentiated into three levels, including elementary education (N = 11,389, 70.4%), secondary education (N = 2,746, 17.0%), and higher education (N = 2,050, 12.7%).
- **Socio-economics (SES)** was replaced by satisfaction of family income level, including low (N = 986, 6.1%), middle-low (N = 3,026, 18.7%), middle (N = 5,552, 34.3%), middle-high (N = 4,878, 30.1%), high (N = 1,742, 10.8%) Since this investigation was conducted according to the unit of community and family. In terms of individual’s report, the satisfaction of family income was more representative especially regarding mental health. Hoebel and colleagues [27] found that lower objective socio-economic status and lower subjective socio-economic status were independently associated with current depressive symptoms and that there was a significant indirect relationship between objective socioeconomic status and depressive symptoms through subjective socioeconomic status.
- **Geography** was clustered into seven regions in China according to the administrative division including North (N = 1,557, 9.6%), Northeast (N = 1,095, 6.8%), East (N = 3,981, 24.6%), Central (N = 2,083, 12.9%), Southwest (N = 1,635, 10.1%), Northwest (N = 2,551, 15.8%), and South (N = 3,283, 20.3%) China.

#### Depressive symptoms

The depressive symptoms were self-reported by using the Chinese edition [28] of the Centre for Epidemiologic Studies Depression (CES-D) Scale [29]. The CES-D is a well-validated screening tool for depression and has been used in many population-based epidemiologic studies worldwide [29, 30]. CES-D contains 20 items about symptoms describing the frequency in a past week on a 4-point Likert scale (ranging from 0 to 3) and the higher marks indicate the severer symptoms. The Chinese version of the CES-D scale has shown acceptable reliability and validity among all age groups both in urban and rural populations. Greater than 15 scores were the recognized value of having depressive symptoms [15, 31, 32]. 17.7% (N = 2858) of individuals were categorized as having depressive symptoms.

#### Birth seasonality

Birth seasonality was categorized based on the common perception of Chinese. Spring is from March to May (N = 3,983, 24.6%), Summer from June to August (N = 4,114, 25.4%), autumn from September to November (N = 4,248, 26.3%), and winter from December to February (N = 3,840, 23.7%). To make the results more comparable, we also selected the representative month for the corresponding birth seasonality, i.e., the middle month of each season. In the secondary analyses, April represented spring, July represented summer, October represented autumn, and January represented winter.

### Statistics

First, univariate chi-square tests were applied to examine the distribution of demographic sub-groups in people having depressive symptoms and people without depressive symptoms. The association between demographic characteristics and depressive symptoms was tested using multiple binary logistic regression. The dummy variable with the lowest proportion of having depressive symptoms in the univariate tests was set as the reference. The estimates used in the model were the odds ratio (OR) and 95% confidence interval (CI). To further investigate distinct associations, four multiple binary logistic regression analyses were carried out in each birth seasonality group. Finally, the interactions between demographic variables and birth seasonality on depressive symptoms were performed.

All statistical tests were performed on IBM Statistic Product and Service Solutions (SPSS) software 24.0 and the significance level was set at 0.05 with two-tailed teston. Prior to the computation of the multiple regression models, we tested the collinearity index, i.e., variance inflation factor (VIF). The test indicated a very low collinearity, as VIF for each variable in each model was less than 2.

## Results

### Primary outcomes

Univariate chi-square tests found that female (χ^2^ = 51.43, *p* < 0.001) and higher age (χ^2^ = 44.80, *p* < 0.001) were significantly associated with having depressive symptoms. The lower SES (χ^2^ = 597.23, *p* < 0.001) and education (χ^2^ = 71.96, *p* < 0.001) were associated with having depressive symptoms. In addition, geography was significantly related to depressive symptoms (χ^2^ = 48.07, *p* < 0.001), of which the rate of having depressive symptoms peaked in Northeast China and bottomed in South China. Regarding seasonality, we observed no significant association with depressive symptoms (χ^2^ = 4.70, *p* > 0.05).

Furthermore, all variables entered the multiple binary logistic regression by setting the minimal proportion of sub-category as the reference (Table 1). The − 2 log likelihood (LL) and receiver operating characteristic (ROC) value of this model were 14,353.67 and 0.65 (95%CI = 0.64, 0.66). We found that the risk factors were female (OR _adjusted_ = 1.38, 95%CI = 1.27, 1.50), old age (> 60 years: OR _adjusted_ = 1.37, 95%CI = 1.17, 1.60), lower SES (low: OR _adjusted_ = 6.13, 95%CI = 5.00, 7.51; middle-low: OR _adjusted_ = 2.91, 95%CI = 2.44, 3.48; middle: OR _adjusted_ = 1.72, 95%CI = 1.45, 2.04; middle-high: OR _adjusted_ = 1.23, 95%CI = 1.03, 1.46), living in other regions than South China (North: OR _adjusted_ = 1.25, 95%CI = 1.06, 1.48; Northeast: OR _adjusted_ = 1.54, 95%CI = 1.28, 1.84; East: OR _adjusted_ = 1.27, 95%CI = 1.12, 1.46; Central: OR _adjusted_ = 1.36, 95%CI = 1.17, 1.58; Southwest: OR _adjusted_ = 1.34, 95%CI = 1.14, 1.57; Northwest: OR _adjusted_ = 1.75, 95%CI = 1.52, 2.01), and born in Summer (OR _adjusted_ = 1.14, 95%CI = 1.02, 1.29).

**Table 1:** Demographic characteristics

Variables	Non-depressive symptoms	Depressive symptoms	Chi-square	OR _adjusted_ (95%CI)
	N (proportion)	N (proportion)		
**Gender**			51.43***	
Male	6,392 (84.6%)	1,160 (15.4%)		Ref
Female	6,935 (80.3%)	1,698 (19.7%)		1.38 (1.27,1.50)***
**Age** (years)			44.80***	
18–30	2,239 (85.5%)	381 (14.5%)		Ref
31–45	3,649 (83.9%)	698 (16.1%)		0.91 (0.79, 1.05)
46–60	5,376 (81.1%)	1,253 (18.9%)		1.13 (0.99, 1.30)
> 60	2,063 (79.7%)	526 (20.3%)		1.37 (1.17, 1.60)***
**Education**			71.96***	
Elementary	9,190 (80.7%)	2,199 (19.3%)		1.14 (0.98, 1.32)
Secondary	2,367 (86.2%)	379 (13.8%)		0.87 (0.73, 1.03)
Higher	1,770 (86.3%)	280 (13.7%)		Ref
**SES**			597.23***	
Low	592 (60.0%)	395 (40.0%)		6.13 (5.00, 7.51)***
Middle-low	2,272 (75.0%)	754 (25.0%)		2.91 (2.44, 3.48)***
Middle	4,652 (83.8%)	900 (16.2%)		1.72 (1.45, 2.04)***
Middle-high	4,256 (87.2%)	622 (12.8%)		1.23 (1.03, 1.46)*
High	1,556 (89.3%)	186 (10.7%)		Ref
**Geography**			48.07***	
South	2,791 (85.0%)	492 (15.0%)		Ref
North	1,282 (82.3%)	275 (17.7%)		1.25 (1.06, 1.48)**
Northeast	871 (79.5%)	224 (20.5%)		1.54 (1.28, 1.84)***
East	3,346 (84.0%)	635 (16.0%)		1.27 (1.12, 1.46)***
Central	1,687 (81.0%)	396 (19.0%)		1.36 (1.17, 1.58)***
Southwest	1,311 (80.2%)	324 (19.8%)		1.34 (1.14, 1.57)***
Northwest	2,039 (79.9%)	512 (20.1%)		1.75 (1.52, 2.01)***
**Birth seasonality**			4.70	
Spring	3,265 (82.0%)	718 (18.0%)		1.11 (0.99, 1.25)
Summer	3,354 (81.5%)	760 (18.5%)		1.14 (1.02, 1.29)*
Autumn	3,536 (83.2%)	713 (16.8%)		Ref
Winter	3,172 (82.6%)	667 (17.4%)		1.06 (0.94, 1.20)

Univariate chi-square tests and multiple logistic regression were shown in the table.

CI: confidence interval; OR: odds ratio; Ref: reference; SES: socio-economics; ***p < 0.001; **p < 0.01; *p < 0.05.

**Fig. 1:**
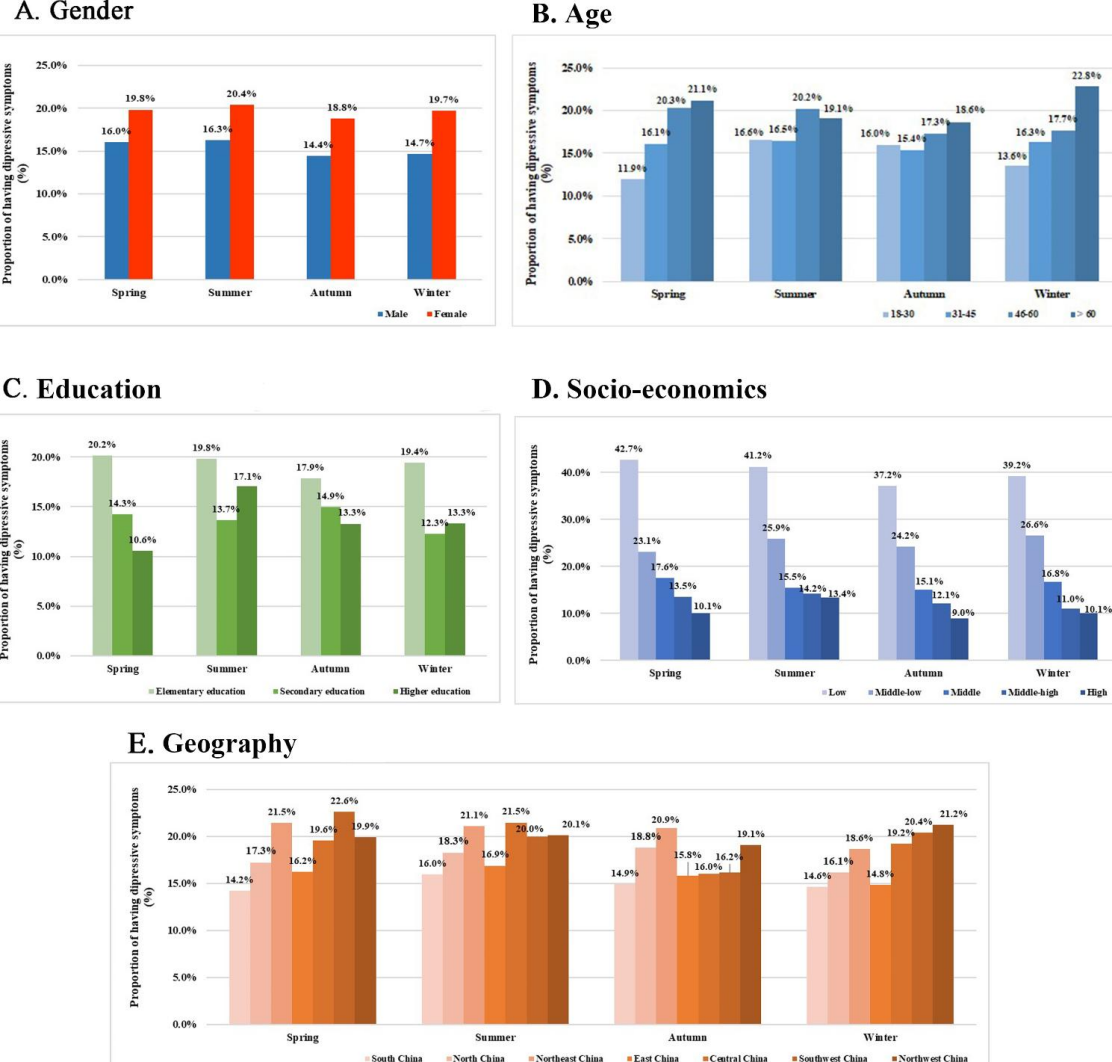
The proportion of having depressive symptoms in individuals with different demographic characteristics across four seasons of birth. (A) Gender and proportion of depressive symptoms; (B) Age and proportion of depressive symptoms; (C) Education and proportion of depressive symptoms; (D) Socio-economics and proportion of depressive symptoms; (E) Geography and proportion of depressive symptoms

Figure 1 illustrates the proportion of having depressive symptoms in individuals with different demographic characteristics across the four birth seasonality groups. The trends for having depressive symptoms in gender and satisfaction of family income were stable across the four seasons of birth (Fig. 1 A **and D**). However, age and education were not stable in birth seasonality in summer (Fig. 1B **and C**), and geography had a different trend for having depressive symptoms among the four seasons of birth (Fig. 1E).

### Multiple binary logistic regression by birth seasonality

Table 2 shows the association between demographic characteristics and depressive symptoms in different birth seasonality groups. The − 2 LL and ROC value of the four models were Spring: 3,546.70 and 0.66 (95%CI = 0.64, 0.68), Summer: 3,764.70 and 0.64 (95%CI = 0.62, 0.66), Autumn: 3,663.35 and 0.65 (95%CI = 0.63, 0.67), and Winter: 3,324.72 and 0.67 (95%CI = 0.65, 0.70).

Compared with males, females were more likely to have depressive symptoms across all birth seasonality groups with OR _adjusted_ ranging from 1.35 to 1.47). Compared to high SES, other sub-groups showed higher likelihood of having depressive symptoms excluding middle-high SES sub-group in all four birth seasonality models.

When compared with 18–31 years group, greater than 60-year-old individuals had a higher likelihood of having depressive symptoms in people born in Spring (OR _adjusted_ = 1.71, 95%CI = 1.23, 2.36) and Winter (OR _adjusted_ = 1.77, 95%CI = 1.27, 2.45). Likewise, for people born in Spring, 46–60 years (vs. 18–31 years) group showed a higher likelihood (OR _adjusted_ = 1.48, 95%CI = 1.11, 1.98) of having depressive symptoms.

For individuals born in Spring, people with elementary education had a higher likelihood of having depressive symptoms compared to people with higher education (OR _adjusted_ = 1.47, 95%CI = 1.05, 2.05). Yet, individuals born in Summer with secondary (vs. higher) education had a lower likelihood of having depressive symptoms (OR _adjusted_ = 0.69, 95%CI = 0.50, 0.96).

Regarding geography, South China was set as the reference. People born in Spring and living in other regions excluding North China, showed a higher likelihood of having depressive symptoms with OR _adjusted_ ranging from 1.35 to 1.95. In people born in Summer, Central (OR _adjusted_ = 1.45, 95%CI = 1.10, 1.94) and Northwest China (OR _adjusted_ = 1.54, 95%CI = 1.17, 2.02) showed higher likelihood. In people born in Autumn, Northeast (OR _adjusted_ = 1.63, 95%CI = 1.14, 2.35) and Northwest China (OR _adjusted_ = 1.69, 95%CI = 1.28, 2.24) showed higher likelihood. In people born in Winter, Central (OR _adjusted_ = 1.41, 95%CI = 1.04, 1.92) and Northwest China (OR _adjusted_ = 1.88, 95%CI = 1.39, 2.54) showed higher likelihood.

**Table 2:** Multiple binary logistic regression by four seasons of birth

Variables	Spring	Summer	Autumn	Winter
	OR _adjusted_ (95%CI)	OR _adjusted_ (95%CI)	OR _adjusted_ (95%CI)	OR _adjusted_ (95%CI)
**Gender** (ref: Male)				
Female	1.35 (1.14, 1.60)***	1.35 (1.14, 1.59)***	1.39 (1.17, 1.64)***	1.47 (1.23, 1.75)***
**Age** (ref: 18–30 y)				
31–45 y	1.08 (0.80, 1.47)	0.81 (0.61, 1.07)	0.80 (0.61, 1.05)	1.00 (0.75, 1.34)
46–60 y	1.48 (1.11, 1.97)**	1.08 (0.83, 1.40)	0.93 (0.71, 1.22)	1.12 (0.84, 1.48)
> 60 y	1.70 (1.23, 2.36)**	1.10 (0.81, 1.51)	1.10 (0.81, 1.50)	1.77 (1.27, 2.45)**
**Education** (ref:Higher)				
Elementary	1.47 (1.05, 2.05)*	0.96 (0.73, 1.26)	1.17 (0.88, 1.56)	1.12 (0.82, 1.51)
Secondary	1.13 (0.77, 1.65)	0.69 (0.50, 0.96)*	1.05 (0.75, 1.47)	0.76 (0.53, 1.09)
**SES** (ref: High)				
Low	7.46 (4.90, 11.36)***	4.98 (3.39, 7.28)***	6.65 (4.37, 10.11)***	6.25 (4.10, 9.52)***
Middle-low	2.74 (1.89, 3.98)***	2.38 (1.72, 3.28)***	3.51 (2.42, 5.08)***	3.31 (2.31, 4.75)***
Middle	2.00 (1.40, 2.84)***	1.25 (0.92, 1.71)	1.94 (1.35, 2.79)***	1.90 (1.35, 2.69)***
Middle-high	1.38 (0.96, 1.98)	1.08 (0.79, 1.49)	1.43 (0.98, 2.07)	1.10 (0.77, 1.58)
**Geography** (ref: South)				
North	1.33 (0.95, 1.87)	1.21 (0.88 1.67)	1.35 (0.98, 1.87)	1.18 (0.82, 1.70)
Northeast	1.84 (1.29, 2.62)***	1.44 (0.98, 2.11)	1.63 (1.14, 2.35)**	1.32 (0.91, 1. 90)
East	1.35 (1.02, 1.78)*	1.27 (0.99, 1.64)	1.28 (0.99, 1.65)	1.22 (0.92, 1.62)
Central	1.51 (1.11, 2.07)**	1.45 (1.09, 1.94)*	1.12 (0.83, 1.51)	1.41 (1.04, 1.92)*
Southwest	1.78 (1.30, 2.45)***	1.25 (0.93, 1.69)	1.06 (0.76, 1.47)	1.38 (0.98, 1.95)
Northwest	1.95 (1.46, 2.60)***	1.54 (1.17, 2.02)**	1.69 (1.28, 2.24)***	1.88 (1.39, 2.54)***

CI: confidence interval; OR: odds ratio; Ref: reference; SES: socio-economics.

***p < 0.001; **p < 0.01; *p < 0.05.

### Interaction of demographic characteristics with birth seasonality

To clarify how the birth seasonality interacts with demographics with respect to depressive symptoms, we performed an interaction analysis (i.e., depressive symptoms “yes/no” = intercept + gender * birth seasonality + age * birth seasonality + education * birth seasonality + SES * birth seasonality + geography * birth seasonality). We set Autumn as the reference, indicating whether same risk factors can be different between Autumn and other three seasons (Table 3). The − 2 LL and ROC value of this model were 14,566.52 and 0.62 (95%CI = 0.61, 0.63).

For gender, we observed the likelihood of females (vs. males) of having depressive symptoms decreased by 20-31% for Autumn-born compared to the other three seasons. Significant results of age were found in the Summer 31–45 years group (OR _adjusted_ = 0.74, 95%CI = 0.57, 0.96) and Winter > 60 years group (OR _adjusted_ = 1.44, 95%CI = 1.07, 1.96). After adding Autumn as the reference, secondary education level significantly reduced the likelihood of having depressive symptoms (Spring: OR _adjusted_ = 0.67, 95%CI = 0.49, 0.91; Summer: OR _adjusted_ = 0.60, 95%CI = 0.44, 0.81; Winter: OR _adjusted_ = 0.55, 95%CI = 0.40, 0.76). Similarly, the association between higher SES and having depressive symptoms was weaker after counting Autumn as the reference. In particular, compared with high SES, low SES (Spring: OR _adjusted_ = 3.86, 95%CI = 2.76, 5.40; Summer: OR _adjusted_ = 4.00, 95%CI = 2.88, 5.57; Winter: OR _adjusted_ = 3.98, 95%CI = 2.79, 5.67) and middle-low SES (Spring: OR _adjusted_ = 1.47, 95%CI = 1.11, 1.95; Summer: OR _adjusted_ = 1.94, 95%CI = 1.49, 2.53; Winter: OR _adjusted_ = 2.13, 95%CI = 1.61, 2.81)induced a higher likelihood of having depressive symptoms. The association between geography and depressive symptoms remained in Northwest (Spring: OR _adjusted_ = 1.37, 95%CI = 1.06, 1.76; Summer: OR _adjusted_ = 1.36, 95%CI = 1.06, 1.74; Winter: OR _adjusted_ = 1.39, 95%CI = 1.07, 1.81) and Central China (Summer: OR _adjusted_ = 1.32, 95%CI = 1.00, 1.73).

**Table 3:** Multiple binary logistic regression of interaction of demographics and birth seasonality (ref: Autumn)

Variables	Spring	Summer	Winter
	OR _adjusted_ (95%CI)	OR _adjusted_ (95%CI)	OR _adjusted_ (95%CI)
**Gender** (ref: Male)			
Female	1.20 (1.02, 1.41)*	1.29 (1.10, 1.51)**	1.31 (1.11, 1.54)**
**Age** (ref: 18–30 y)			
31–45 y	0.80 (0.61, 1.05)	0.74 (0.57, 0.96) *	0.80 (0.62, 1.05)
46–60 y	1.14 (0.89, 1.47)	1.00 (0.78, 1.28)	0.94 (0.72, 1.21)
> 60 y	1.28 (0.96, 1.72)	1.01 (0.75, 1.37)	1.44 (1.07, 1.96)*
**Education** (ref: Higher)			
Elementary	0.93 (0.71, 1.20)	0.85 (0.66, 1.09)	0.85 (0.66, 1.10)
Secondary	0.67 (0.49, 0.91)*	0.60 (0.44, 0.81)**	0.55 (0.40, 0.76)***
**SES** (ref: high)			
Low	3.86 (2.76, 5.40)***	4.00 (2.88, 5.57)***	3.98 (2.79, 5.67)***
Middle-low	1.47 (1.11, 1.95)**	1.94 (1.49, 2.53)***	2.13 (1.61, 2.81)***
Middle	1.04 (0.81, 1.33)	1.01 (0.79, 1.29)	1.18 (0.91, 1.52)
Middle-high	0.74 (0.57, 0.96)*	0.88 (0.68, 1.13)	0.69 (0.53, 0.92)*
**Geography** (ref: South)			
North	0.97 (0.71, 1.33)	1.09 (0.80 1.47)	0.88 (0.63, 1.23)
Northeast	1.31 (0.94, 1.82)	1.28 (0.89, 1.84)	1.00 (0.71, 1.41)
East	0.98 (0.77, 1.26)	1.13 (0.90, 1.43)	0.92 (0.72, 1.18)
Central	1.12 (0.84, 1.49)	1.32 (1.00, 1.73)*	1.09 (0.83, 1.45)
Southwest	1.34 (1.00, 1.80)*	1.15 (0.87, 1.53)	1.10 (0.80, 1.52)
Northwest	1.37 (1.06, 1.76)*	1.36 (1.06, 1.74)*	1.39 (1.07, 1.81)*

CI: confidence interval; OR: odds ratio; Ref: reference; SES: socio-economics.

***p < 0.001: **p < 0.01; *p < 0.05.

## Discussion

China spans over an enormous geographical area, making the country extremely diverse in terms of both population and climate [26]. For example, there is a great seasonal and regional difference with the former affecting early life by interfering with the mood and hormonal level of pregnant women [33–35]. In the same vein, the food supply and dietary pattern also change with the seasons [36–38]. As a result, seasons may influence the development of individuals in early life. Particularly, some papers have highlighted the effects of birth seasonality on depressive symptoms. In addition, the development of depressive symptoms has shown to be more prevalent in certain vulnerable demographic groups. Hence, the present study explored the influence of birth seasonality on depressive symptoms in adults through demographic factors. Consistent with previous literature [39, 40], we found that females, elders, low education, low socio-economics, and living in the North China were the significant risk factors of having depressive symptoms. Moreover, people born in Autumn had the lowest rate (16.8%) of having depressive symptoms. Compared with people born in Autumn, people born in Summer exhibited a higher likelihood (OR = 1.14, 95%CI = 1.02, 1.29) of having depressive symptoms when controlling for demographic variables. In particular, gender, SES, and education were consistent in having depressive symptoms but age or geography were not.

First, gender and SES, both of which are associated with depressive symptoms across birth seasonality, might be less influenced by the environment. In line with previous studies [41, 42], females showed a higher likelihood of having depressive symptoms than male individuals. Female individuals often experience a so-called “sandwiched life” between household and work [43, 44]. Besides, women tend to be more emotionally sensitive when facing such problems [45]. Hence, females endure much more pressure in daily life. Besides, SES was also strongly associated with adulthood depressive symptoms across birth seasonality. A large number of surveys has similarly reported that lower-income level is an important risk factor in adulthood depressive symptoms, particularly in the Asian countries [46]. Due to economic pressure from childcare and daily expenses among other things, many adults become anxious about their financial status. As a subject financial factor, SES cannot only reflect one’s financial status but also self-evaluated society status [47, 48]. Our results suggested that satisfaction of family income status plays a key role in having depressive symptoms. As for education, we found that higher educated individuals showed lower likelihood of having depressive symptoms. The results were consistent with the previous study from China [39].

However, age-depressive symptoms association can change across different birth seasonality groups. Only for people born in Spring and Winter, age was significantly related to depressive symptoms. Mainly in 46–60 or > 60 years old sub-groups, the difference of age on depressive symptoms across four seasons of birth groups was significant. Those people were mainly born in an era of material deprivation, and their standard of living was relatively low. Besides, the refrigerator has not yet entered the ordinary family household, which implied that food could not be well preserved. The seasons might therefore affect the food supply in people’s daily life. The shortage of food in winter and early spring might greatly influence maternal nutrition. The weather is relatively cold and in winter and early spring in China. Cold weather may influence the health status of pregnant women in the 1960s. Besides, seasonal infectious diseases in spring may also affect the health status of individuals. The birth season effects in adulthood depression at younger individuals are not significant. It might be attributed to the booming of economy and development of technology. These factors might explain the increased likelihood of having depressive symptoms among people born in Spring and Winter.

As a categorical variable, geography had different associations with depressive symptoms across birth seasonality. Compared with Southwest China, people in the Northeast and Northwest China were more likely to be depressed. The regional climate is commonly dryer and colder than South China. Low humidity and cold weather have been demonstrated as the possible risk factors of depression [49, 50]. The sensitivity of geography on adulthood depression was changed by birth seasonality. Compared with people born in autumn, individuals born in spring and summer generally showed higher likelihood of having depression. This phenomenon further supported our hypothesis.

Univariate analysis found that birth seasonality was not significantly related to adulthood depressive symptoms, yet significant after controlling for demographic variables. Previous reports mainly focused on the relationship between birth seasonality on physical diseases or severe mental disorders like schizophrenia [26, 51, 52]. In addition, most studies investigating the effects of birth seasonality on depression were from a patient population with a relatively modest number of samples [23, 53]. More importantly, the trends of adulthood depressive symptoms in different seasons of birth have seldom been reported from China. Compared with the previous studies, the sampling method was more scientific and systemic in our work. In particular, we investigated individuals from 29 provinces in China with a valid number of 16,185. The population in the present research was therefore large and representative.

However, there are several drawbacks in our work. First, the present study mainly investigated individuals from the labor-forces in China and the associations we aimed to study were somehow weak. Secondly, the respondent rate reaches 76.7% and most non-respondents are due to missing date of birth. Such non-respondents may cause subtle bias but comparing the depression rate, gender ratio, and age ratio between respondents and full sample [12] suggests that the current sample still remains representative. Although laborers are the majority in the population, it still limits the extrapolation of our conclusions to the general population. Hence, further study may focus on comparing the birth seasonality effects across different populations. Many scholars have put forwarded possible explanations about the effects of birth season on depression, such as maternal nutrition, seasonal infectious diseases, genetics, and hormone levels [23, 54–56]. Nevertheless, the real mechanisms of how the birth seasonality affects depressive symptoms remain unclear. Stronger shreds of evidence may need animal experiments or genetic research about the effects of birth seasonality on depression. Lastly, the investigation of the present work was cross-sectional and conducted in Summer, meaning that causal explanations are questionable and limited to investigation in Summer. To testify the influence of birth seasonality on demographic factors of depression, future studies should optimally include a more representative sample of the general population in a longitudinal design measured across four seasons.

## Conclusion

To summarize, we found that birth seasonality had a significant influence on depressive symptoms when controlling for demographic variables. Compared with people born in Autumn, people born in Summer showed a higher likelihood of having depressive symptoms (OR = 1.14, 95%CI = 1.02, 1.29). In different birth seasonality groups, the association between demographic variables and depressive symptoms was different especially for age and geography. Since a large body of evidence has highlighted the current climate effects on depressive symptoms, our findings further explored how rough early life climate environment contributes to adulthood depression. Conclusively, this work provides new insights about the climate effects on depressive symptoms and may help guide environment-targeted preventive policies.

## Data Availability

Data analyzed in this manuscript were obtained from the CLDS project. The raw data may not be shared by third parties due to ethics requirements but can be obtained via contacting the Center for Social Survey at Sun Yat-sen University [cssdata@mail.sysu.edu.cn].

## References

[1] Hsieh CR, Qin X (2018). Depression hurts, depression costs: the medical spending attributable to depression and depressive symptoms in China. Health Econ.

[2] James SL, Abate D, Abate KH (2018). Global, regional, and national incidence, prevalence, and years lived with disability for 354 diseases and injuries for 195 countries and territories, 1990–2017: a systematic analysis for the global burden of Disease Study 2017. The Lancet.

[3] Herrman H, Kieling C, McGorry P (2019). Reducing the global burden of depression: a Lancet-World Psychiatric Association Commission. Lancet.

[4] Cao Y, Li W, Shen J (2013). Health-related quality of life and symptom severity in chinese patients with major depressive disorder. Asia Pac Psychiatry.

[5] Zhang Y, Chen Y, Ma L (2018). Depression and cardiovascular disease in elderly: current understanding. J Clin Neurosci.

[6] You L, Yu Z, Zhang X (2019). Association between Multimorbidity and Depressive Symptom among Community-Dwelling Elders in Eastern China. Clin Interv Aging.

[7] Murakami H, Shiraishi T, Umehara T (2020). Differences in correlations of depression and anhedonia with cardiovascular sympathetic functions during a head-up tilt test in drug-naive Parkinson’s disease patients. Neurol Sci.

[8] Ren X, Yu S, Dong W (2020). Burden of depression in China, 1990–2017: findings from the global burden of disease study 2017. J Affect Disord.

[9] Ru J, Ma J, Niu H (2019). Burden and depression in caregivers of patients with rheumatoid arthritis in China. Int J Rheum Dis.

[10] Guo Y, Sun J, Hu S, et al. Hospitalization costs and financial burden on families with children with Depression: a cross-section study in Shandong Province, China. Int J Environ Res Public Health. 2019;16(19). 10.3390/ijerph16193526. 10.3390/ijerph16193526 PMC6801864 31547207

[11] Nolen-Hoeksema S, Larson J, Grayson C (1999). Explaining the gender difference in depressive symptoms. J Pers Soc Psychol.

[12] Xu CJ, Wu WJ, Peng-Li D (2020). Intraday weather conditions can influence self-report of depressive symptoms. J Psychiatr Res.

[13] Alonso Debreczeni F, Bailey PE (2020). A systematic review and Meta-analysis of subjective age and the Association with Cognition, subjective wellbeing, and Depression. J Gerontol B Psychol Sci Soc Sci.

[14] Pei YL, Cong Z, Wu B. (2020) Education, adult children’s education, and depressive symptoms among older adults in rural China.Soc Sci Med253 10.1016/j.socscimed.2020.112966 32247217

[15] Pan A, Franco OH, Wang Y-f (2008). Prevalence and geographic disparity of depressive symptoms among middle-aged and elderly in China. J Affect Disord.

[16] Agarwal N, Aiyar A, Bhattacharjee A (2017). Month of birth and child height in 40 countries. Econ Lett.

[17] Douros K, Fytanidis G, Papadimitriou A (2019). Effect of the month of birth on the height of young adult males. Am J Phys Anthropol.

[18] Sohn K (2015). The influence of birth season on height: evidence from Indonesia. Am J Phys Anthropol.

[19] Torrey EF, Rawlings RR, Ennis JM (1996). Birth seasonality in bipolar disorder, schizophrenia, schizoaffective disorder and stillbirths. Schizophr Res.

[20] Tonetti L, Fabbri M, Martoni M (2012). Season of birth and mood seasonality in late childhood and adolescence. Psychiatry Res.

[21] Talarowska M, Blizniewska K, Wargacka K (2018). Birth Month and Course of Recurrent Depressive Disorders in a Polish Population. Med Sci Monit.

[22] Tonetti L, Fabbri M, Natale V (2009). Season of birth and personality in healthy young adults. Neurosci Lett.

[23] Mino Y, Oshima I, Okagami K (2000). Seasonality of birth in patients with mood disorders in Japan. J Affect Disord.

[24] Björkstén KS, Bjerregaard P (2015). Season of birth is different in Inuit suicide victims born into traditional than into modern lifestyle: a register study from Greenland. BMC Psychiatry.

[25] Joiner TE, Pfaff JJ, Acres JG (2002). Birth month and suicidal and depressive symptoms in Australians born in the Southern vs. the Northern hemisphere. Psychiatry Res.

[26] Wang C, Zhang Y (2017). Season of birth and schizophrenia: evidence from China. Psychiatry Res.

[27] Hoebel J, Maske UE, Zeeb H (2017). Social Inequalities and depressive symptoms in adults: the role of objective and subjective socioeconomic status. PLoS ONE.

[28] Zhang J, Wu Z, Fang G (2010). Development of the chinese age norms of CES-D in urban area. Chin Mental Health J.

[29] Radloff LS (1977). The CES-D scale: a self-report Depression Scale for Research in the General Population. Appl Psychol Meas.

[30] Radloff LS (1991). The use of the Center for epidemiologic Studies Depression Scale in adolescents and young adults. J Youth Adolesc.

[31] Hong Y, Li X, Fang X (2007). Depressive symptoms and condom use with clients among female sex workers in China. Sex Health.

[32] Gu ZH, Qiu T, Tian FQ (2020). Perceived Organizational Support Associated with depressive symptoms among Petroleum Workers in China: a cross-sectional study. Psychol Res Behav Manag.

[33] Ismailova K, Poudel P, Parlesak A (2019). Vitamin D in early life and later risk of multiple sclerosis-A systematic review, meta-analysis. PLoS ONE.

[34] Luykx JJ, Bakker SC, Lentjes E (2012). Season of sampling and season of birth influence serotonin metabolite levels in human cerebrospinal fluid. PLoS ONE.

[35] Tonetti L, Milfont TL, Tilyard BA (2013). Month of birth and mood seasonality: a comparison between countries in the northern and southern hemispheres. Psychiatry Clin Neurosci.

[36] Stelmach-Mardas M, Kleiser C, Uzhova I (2016). Seasonality of food groups and total energy intake: a systematic review and meta-analysis. Eur J Clin Nutr.

[37] Zhang QF, Lyu CP, Zhou JC (2019). [Nutrient and metabolic responses of the leaves of Cunninghamia lanceolata seedlings to warming and reduced precipitation in different season]. Ying Yong Sheng Tai Xue Bao.

[38] Zhu Z, Wu C, Luo B (2019). The Dietary Intake and its features across Four Seasons in the Metropolis of China. J Nutr Sci Vitaminol (Tokyo).

[39] Qin X, Wang S, Hsieh C-R (2018). The prevalence of depression and depressive symptoms among adults in China: Estimation based on a National Household Survey. China Econ Rev.

[40] Wu H, Li H, Ding Y (2020). Is triglyceride associated with adult depressive symptoms? A big sample cross-sectional study from the rural areas of central China. J Affect Disord.

[41] Salk RH, Hyde JS, Abramson LY (2017). Gender differences in depression in representative national samples: Meta-analyses of diagnoses and symptoms. Psychol Bull.

[42] Culbertson FM (1997). Depression and gender. An international review. Am Psychol.

[43] Parker G, Brotchie H (2010). Gender differences in depression. Int Rev Psychiatry.

[44] Borooah VK (2010). Gender differences in the incidence of depression and anxiety: Econometric evidence from the USA. J Happiness Stud.

[45] Vafaei A, Ahmed T, Freire Ado N (2016). Depression, sex and gender roles in older adult populations: the International mobility in Aging Study (IMIAS). PLoS ONE.

[46] Gero K, Kondo K, Kondo N (2017). Associations of relative deprivation and income rank with depressive symptoms among older adults in Japan. Soc Sci Med.

[47] Hoebel J, Muters S, Kuntz B (2015). Measuring subjective social status in health research with a german version of the MacArthur Scale. Bundesgesundheitsbla.

[48] Reitzel LR, Vidrine JI, Li YS (2007). The influence of subjective social status on vulnerability to postpartum smoking among young pregnant women. Am J Public Health.

[49] Davis RE, McGregor GR, Enfield KB. Humidity: a review and primer on atmospheric moisture and human health. Environ Res 144 (Pt A). 2016;106–16. 10.1016/j.envres.2015.10.014. 10.1016/j.envres.2015.10.014 26599589

[50] Ding N, Berry HL, Bennett CM (2016). The importance of humidity in the relationship between Heat and Population Mental Health: evidence from Australia. PLoS ONE.

[51] Troisi A, Pasini A, Spalletta G (2001). Season of birth, gender and negative symptoms in schizophrenia. Eur Psychiatry.

[52] Soreca I, Cheng Y, Frank E (2013). Season of birth is associated with adult body mass index in patients with bipolar disorder. Chronobiol Int.

[53] Park SC, Sakong JK, Koo BH (2016). Potential relationship between season of birth and clinical characteristics in major depressive disorder in Koreans: results from the CRESCEND Study. Yonsei Med J.

[54] Schnittker J (2018). Season of birth and depression in adulthood: revisiting historical forerunner evidence for in-utero effects. SSM - population health.

[55] Chotai J, Serretti A, Lattuada E (2003). Gene-environment interaction in psychiatric disorders as indicated by season of birth variations in tryptophan hydroxylase (TPH), serotonin transporter (5-HTTLPR) and dopamine receptor (DRD4) gene polymorphisms. Psychiatry Res.

[56] Levitan RD, Masellis M, Lam RW (2006). A birth-season/DRD4 gene interaction predicts weight gain and obesity in women with seasonal affective disorder: a seasonal thrifty phenotype hypothesis. Neuropsychopharmacology.

